# Editorial: Therapeutic drug monitoring (TDM): a useful tool for pediatric pharmacology applied to routine clinical practice, Volume II

**DOI:** 10.3389/fphar.2023.1250784

**Published:** 2023-07-25

**Authors:** Raffaele Simeoli

**Affiliations:** Division of Metabolic Diseases and Drug Biology, Bambino Gesù Children’s Hospital, IRCCS, Rome, Italy

**Keywords:** therapeutic drug monitoring, pediatrics, pharmacokinetic/pharmacodynamics, popPK, microsampling

## 1 Background

The annual meeting of International Association of TDM and Clinical Toxicology (IATDMCT), held in Prague on September 2022, has offered several and significant discussion points. Among them, the debate TCI (target concentration intervention) vs*.* TDM (therapeutic drug monitoring) has aroused the interest of many attenders due to the great actuality that characterizes this theme. The concept of TCI was explained by Holford et al. (2022) in a review published on the *British Journal of Clinical Pharmacology*. According to the authors, TCI has a single target concentration that allows to calculate the most indicated dose for each patient. Conversely, TDM provides a range of concentrations defined as “therapeutic range”. The authors provide detailed arguments on the main differences between TCI and TDM, declaring that in a personalized medicine context, TCI should be preferred over TDM as standard strategy for dose individualization. Finally, they conclude that both scientific principles and clinical evidence seem to suggest TCI as the ideal treatment approach.

On the other side, a commentary made by Lemaitre F. and Hesselink D. A. (2023) provides a point by point rebuttal to Holford’s article and affirms that TDM is not dead but is alive and kicking. In particular, the authors declare that in current clinical practice the difference between TCI and TDM is mostly based on a traditional and simplified view of TDM. In fact, TDM is actually more than a “simple” drug concentration measurement in body fluids. Therefore, Lemaitre F. and Hesselink D. A conclude that TCI concept could be mostly defined as TDM in a “new bag”. In this *scenario,* TDM and TCI should not be considered as enemies but only faces of the same medal.

Nowadays, the introduction of new microsampling devices and the spread of pharmacometric methods, including population (popPK) and physiologically based (PBPK) pharmacokinetics models, has aroused an increasing interest towards TDM. As consequence, TDM is not only applied to drugs for whom is considered mandatory but represents a useful tool for exploring the PK behavior of different medications even in special populations (i.e., neonatal and pediatric population). In fact, bioanalytical procedures are not always applicable due to the limited sampling volumes collected in neonates and children, thereby limiting the application of TDM in clinical practice. Additionally, ethical aspects represent an important issue in conducing PK studies on such special population. However, despite the advantages offered by microsampling procedures and modelling approaches, knowledge of TDM among clinicians is still theoretical and its application during clinical practice in not close to a routinary use.

## 2 Scope

This article Research Topic is directly connected to the first edition (Volume I) published 1 year ago. As for Volume I, in which we have collected manuscripts focused on antimicrobials, immunosuppressants, neonatal TDM and bioanalytical methods applied to TDM, in this second Volume we aimed to receive contributes that describe the utility of applying TDM practice in pediatric patients and in different disease settings. Similar to the previous edition, also for the second volume of this Research Topic we have collected both reviews and original articles showing the application of TDM-associated PK/PD models and microsampling techniques to dose optimization strategies in pediatric subjects including neonates and critically ill children.

## 3 Overview of contributions

### 3.1 Immunosuppressant therapy

TDM is widely used for guiding immunosuppressive therapy and is routinely applied to calcineurin- and mTOR inhibitors. A recent Research Topic focusing on TDM practice in organ transplantation procedures has nicely collected nine original articles highlighting the importance of performing TDM for immunosuppressive drugs and its utility in providing individualized recommendations (Staatz et al., 2021). So far, several consensus guidelines have provided optimal TDM instructions for these agents (Shipkova et al., 2016; Bergan et al., 2021).

In our previous article Research Topic, we have received a study describing a popPK model for tacrolimus given as intravenous continuous infusion in pediatric and young adult patients subjected to hematopoietic stem cell transplantation (HSCT) (Brooks et al., 2021). Here, a review by Shen et al. shows that, although TDM of sirolimus at trough levels (before the next dose) is the actual standard of care, C*min* are modestly correlated with the area under the curve (AUC). As consequence, despite the use of TDM for sirolimus, is not surprisingly that patients treated with this drug are characterized by high extent variability in PK parameters, efficacy results and adverse events appearance. The authors, therefore, conclude that a model-informed precision dosing (MIPD) could be useful and should be improved, whilst there are not enough data to support DBS as point–of–care sampling for silorimus precision dosing. However, future studies should include pharmacogenomic and pharmacometabolomic approaches to predict sirolimus PK and to evaluate point-of-care determination of drug levels in blood, saliva, or sweat Shen et al.

### 3.2 Antimicrobial therapy

Usually, neonatal pharmacology extends similar considerations to preterm and full-term newborns. However, preterms are characterized by physiological and pharmacological properties that make them different from full-terms and, therefore, require specific evaluations (Allegaert et al., 2007). Antibiotics represent a drug class widely used among preterms. In fact, during their hospitalization in neonatal intensive care units (NICUs), central catheters and ventilators are often vehicles of infections. However, despite the wide use of antibiotics among neonatal population, preterms are rarely involved in clinical trials, determining a gap of knowledge on the PK behavior of most drugs used among these patients. Therefore, microsampling procedures and TDM-associated popPK studies could be useful to overcome this limit and to fill the lack of knowledge.

Meropenem is one of the most used carbapenem among newborns, especially for treatment of late-onset sepsis (LOS) and complicated intra-abdominal infections (Cohen-Wolkowiez et al., 2012;Hussain et al., 2021). A study conducted by Zyryanov et al. aimed to realize a TDM-based popPK study on the use of meropenem in preterm infants and to evaluate the pharmacodynamics (PD) indices as well as covariates affecting the PK parameters. In this study, meropenem concentrations were determined by high-performance liquid chromatography (HPLC) in 132 samples from *n* = 66 preterm newborns. Gestational age (GA), postnatal age (PNA), post-conceptual age (PCA), body weight (BW) and creatinine clearance were evaluated as covariates influencing PK parameters. The univariate regression analysis showed that creatinine clearance, BW and PCA were the main covariates influencing half-life (T1/2) whereas meropenem distribution volume (Vd) was mainly affected by BW and PCA. Therefore, the authors conclude that a model-based approach together with TDM data could be useful in personalizing meropenem dosing strategies. In fact, this popPK model can be used as Bayesian prior information to estimate PK parameters in preterms and to predict the desired PK/PD target attainment once TDM results are available Zyryanov et al.

Among glycopeptides antibiotics, vancomycin is used in newborns to treat severe infections including late-onset sepsis caused by Gram-positive bacteria, such as methicillin-resistant *Staphylococcus aureus* and coagulase negative staphylococci (CoNS) (Zeng et al., 2016). In terms of PD targets, a study performed by Samb et al. has proposed bacterial DNA loads (BDL) as surrogate marker to evaluate vancomycin efficacy in preterms and very low birth weight (VLBW) newborns with late-onset sepsis. In fact, whilst LOS diagnosis is currently based on positive blood cultures, these analyses can require days and earlier indices of treatment efficacy are missing. Therefore, the authors have proposed to investigate whether efficacy of vancomycin treatment could be evaluated using bacterial DNA loads determined by real-time quantitative polymerase chain reaction (RT-qPCR). To this aim, they have performed a population PK/PD modeling by using non-linear mixed-effects modeling (NONMEM) software. Twenty-eight patients affected by LOS and treated with vancomycin were included, whilst a one-compartment model with post-menstrual age (PMA) and weight as main covariates was adopted to depict the PK profile vs*.* time of vancomycin. Results showed a linear correlation between vancomycin concentration and first-order BDL elimination. In *n* = 12 patients for whom no decrease in BDL over time was observed, a clinical non-response was reported. Therefore, the authors conclude that RT-qPCR analyses of BDLs were sufficiently described by the developed popPK model. Moreover, evaluation of clinical response to vancomycin by using BDL in LOS can be verified within 8 h after treatment beginning Samb et al.

DBS is widely used in neonatal screening programs and blood home sampling (Delahaye et al., 2021). Recent evidence has highlighted an increasing attention on DBS for quantitative applications, including TDM (Morgan, 2021). However, drug concentrations measured on DBS could be over- or underestimated in samples if the hematocrit (Hct) value significantly differs from the blood used for preparing calibrators (Morgan, 2021). Moreover, although microsampling devices could facilitate TDM application to neonates and children, these tools should be opportunely validated by comparing drugs’ concentrations in capillary vs*.* venous EDTA whole blood samples.

Xu et al. have conducted a pilot study in order to develop and cross-validate dried blood spots (DBS) and capillary microsamples (CMS) as microsampling strategies for TDM of vancomycin, meropenem and linezolid in critically ill children. Deming regression and Bland–Altman tests were used to cross-validate methods. These results showed for both DBS and CMS an accuracy and precision value within the acceptable ranges. A positive and significant correlation was found between CMS and DBS antibiotic concentrations (r > 0.98). Moreover, DBS rather than CMS were slightly influenced by hematocrit values. However, EPC (estimated plasma concentrations) were calculated from the DBS by using single hematocrit ranges. The EPC showed comparable results for vancomycin (slope: 1.06) and meropenem (slope: 1.04), with an average of 98% and 99% of the measured CMS values, respectively. Therefore, the authors conclude that DBS and CMS could represent a valid microsampling alternative to be used during routine practice. Additionally, both devices could be adopted within a TDM-based approach to optimize antimicrobial therapy in critically ill children, overcoming the limitations on the number and volumes of blood samples withdrawable in pediatric patients Xu et al.

### 3.3 TDM of antiepileptic drugs (AEDs)

Despite the absence of randomized studies showing a positive effect of TDM on clinical outcome in epileptic patients, data available from nonrandomized studies and real-life clinical practice seem to suggest that monitoring plasma concentrations of antiepileptic drugs (AEDs) can be useful in guiding patient management. Moreover, variability in PK behavior including non-linear metabolism or drug-drug interactions (DDIs) strengthen the application of TDM to this class of drugs (Johannessen Landmark et al., 2020;Fluckiger et al., 2022). The International League Against Epilepsy (ILAE) has provided guidelines for TDM (Patsalos et al., 2008). In particular, ILAE guidelines describe also those clinical settings in which AEDs monitoring could be useful and beneficial (Patsalos et al., 2008). Lacosamide (LCM) is a new generation anti-seizure medication (AM) endowed with linear PK, predictable plasma concentrations and limited DDIs (Schultz and Mahmoud, 2020). Compared to older generation AMs such as phenytoin and carbamazepine for whom TDM is strongly advised, necessity of monitoring plasma concentration for lacosamide is still debated (Schultz and Mahmoud, 2020). However, some reports have shown variable PK profiles for LCM in different patient populations suggesting the necessity of TDM (Schultz and Mahmoud, 2020). The utility of TDM for LCM in pediatric patients with drug resistant epilepsy has been investigated in a retrospective observational study conducted by Kohn et al. In this study, demographic and clinical data were extracted from medical records of children with refractory epilepsy under treatment with LCM at Shamir Medical Center (Tel Aviv, Israel) between February 2019 and September 2021. Database included only patients for whom LCM trough levels were available. Totally, *n* = 42 children (range age: 1–18 years) were included in the study. The average of LCM serum concentration was 6.74 ± 3.27 mg/L (mean ± SD). Correlations between LCM serum levels and clinical response (*p* = 0.58), and between LCM dosage and change in seizure rate (*p* = 0.30) did not show significant associations. Therefore, the authors conclude that based on the results of this observational study, TDM of lacosamide is not needed in all children treated with this drug. Finally, they propose that measuring serum concentrations may be useful in patients with refractory epilepsy in order to evaluate therapy adhesion or in patients with adequate control of seizures to establish their therapeutic basal level Kohn et al.

## 4 Conclusion

In the debate between TCI vs*.* TDM, a famous sentence could summarize as follow: “In medio stat virtus” (virtue lies in the middle). However, in contrast to the explanation provided for this sentence by the philosopher Aristotele in his manuscript “*Etica Nicomachea”,* TCI and TDM are not opposites to be equally avoided but two faces of the same medal. In fact, nowadays TDM is more than a “simple” drug concentration measurement in body fluids, and the “TCI concept” does not seem to introduce evident changes. Therefore, concepts of TCI and TDM could integrate each other and according to the definition provided by Lemaitre F. and Hesselink D. (2023), “TCI mostly is TDM in a new bag”.

In this Research Topic, we aimed to support TDM for tailoring pharmacological treatments in pediatric patients including neonates and critically ill children. Manuscripts published within this article Research Topic highlight how TDM-guided popPK and microsampling devices could be a valid tool for TDM application. In fact, despite last decades have shown a TDM implementation toward different drug’s classes, its introduction into clinical practice is still far from a routine *scenario*. Similarly, a growing interest has emerged toward the use of model-informed precision dosing and TDM-guided popPK studies. These approaches, combined to innovative microsampling techniques, could represent a valid strategy to overcome the ethical concerns that often limit TDM practice in pediatric patients and the involvement of these subjects in PK studies. In conclusions, we believe that articles presented within this Research Topic focus on two important topics that could significantly implement TDM application in the routine clinical practice.
